# Beyond the Usual Suspects: Hereditary Hemochromatosis and Transaminitis in Primary Care

**DOI:** 10.7759/cureus.43481

**Published:** 2023-08-14

**Authors:** Venkata Sri Ramani Peesapati, Paavana Varanasi, Harish Patel, Sai Lakshmi Akella

**Affiliations:** 1 Internal Medicine, BronxCare Health System, New York, USA; 2 Internal Medicine, BronxCare Health System, Bronx, USA; 3 Medicine/Gastroenterology, BronxCare Health System, Bronx, USA; 4 General Medicine, BronxCare Health System, Bronx, USA

**Keywords:** annual physical exam, uncontrolled dm, hfe gene mutation, primary care, phlebotomy, elevated ferritin level, alcohol consumption, hereditary hemochromatosis, fatty liver, transaminits

## Abstract

An annual physical examination within a primary care setting, including evaluation of liver enzymes and abnormal serology, is incidental and often asymptomatic. Fatty liver is the most common etiology for transaminitis. Hepatobiliary imaging studies, viral hepatitis serology, evaluation of metabolic liver disease, and alcohol consumption history should be performed for transaminitis evaluation. In patients with prior history of excessive alcohol consumption, transaminitis is often assumed to be alcohol-related. It is prudent to evaluate other infectious and metabolic etiologies, which can change patient management. Iron studies, including ferritin and transferrin saturation, are performed to evaluate hereditary hemochromatosis (HH). We present the case of a 46-year-old patient who visited the clinic for a routine health checkup, during which elevated ferritin levels were detected. Subsequent diagnosis revealed hemochromatosis. The patient underwent phlebotomy, resulting in a reduction of ferritin levels.

## Introduction

Hereditary hemochromatosis (HH) is rare and likely underdiagnosed. When diagnosed at later stages, HH is characterized by the triad of liver cirrhosis, hyperpigmentation, and diabetes, often referred to as *bronze diabetes* [1,2]. HH is frequently caused by two mutations, C282Y (cysteine to tyrosine substitution at amino acid 282) and H63D (histidine-to-aspartic acid substitution at amino acid 63). Although H63D high iron gene (HFE) mutation is associated with hemochromatosis, fewer patients present with evidence of iron load despite elevated ferritin levels. Many patients with hemochromatosis, however, do have a diagnosis of diabetes mellitus. The pathogenesis involves damage to beta cells and secondary insulin resistance due to the deposition of iron in the pancreas.

## Case presentation

A 46-year-old Hispanic male presented to our clinic for his annual physical exam. On presentation, the patient was hypertensive, the blood pressure was 151/107 mmHg, pulse was 103 beats per minute, and temperature was 98.7 °F. His medical history was significant for hypertension, uncontrolled diabetes mellitus type 2, hyperlipidemia, obesity (body mass index [BMI] 31.8), and alcohol dependence. The physical examination revealed an obese male, but his review of systems was unremarkable. Family history was negative for any hepatic disorder but positive for hypertension and diabetes mellitus. His social history was significant for smoking cigarettes two to three times per week for more than 10 years and drinking whiskey more than 20 drinks per week for the last 30 years, but he denied illicit drug use. Urine toxicology was negative. His home medications were losartan 50 mg for hypertension, atorvastatin 40 mg, metformin 1,000 mg every 12 hours, and glipizide 5 mg extended release for diabetes.

The patient was initially seen in the clinic in 2021 after hospital admission for diabetic ketoacidosis. Later, he was lost to follow-up until he presented in November 2022. Laboratory evaluation was significant for elevated hemoglobin of 18 g/dL, with the mean corpuscular volume (MCV) of 104.8 fL, transaminitis with the alanine aminotransferase (ALT) and aspartate aminotransferase (AST) levels of 50 and 36 units/L, respectively, and a normal white blood count. As the patient had an elevated ferritin level in 2021, iron studies were repeated when he presented to the clinic in 2022. His serum iron and ferritin were 223 microg/dL and 998 microg/mL with a transferrin saturation of 69% (Table 1). The patient was referred to a gastroenterology clinic for transaminitis and a hematology clinic for polycythemia and high ferritin levels. 

**Table 1:** Laboratory investigations. AST, aspartate aminotransferase; ALT, alanine aminotransferase; Alk Phos, alkaline phosphate transferase; Tot. bilirubin, total bilirubin; UIBC, unsaturated iron binding capacity; Tsat, transferrin saturation; N/A, not available

Timeline	One year prior	On presentation	Repeat labs after one week	Reference range
AST	62	36	36	9-48 unit/L
ALT	91	50	50	5-40 unit/L
Alk Phos	143	158	158	53-128 unit/L
Tot. bilirubin	0.6	0.4	0.4	0.2-1.2 mg/dL
Direct bilirubin	0.3	0.4	0.2	0.0-0.3 mg/dL
Iron	N/A	223	187	65-175 microg/dL
UIBC	N/A	101	132	112-346 microg/dL
Tsat	N/A	69%	141.67%	15%-50%
Ferritin	2,582	998	1,292	13-150 ng/mL
Hemoglobin	15	18.2	17.4	12-16 g/dL
Hematocrit	43.2	53.9	51.5	42%-51%

We pursued further investigations to evaluate for transaminitis. His celiac serology and viral hepatitis panel were unrevealing. The anti-mitochondrial antibody, smooth muscle antibody, and ceruloplasmin levels were normal. His ultrasound of the abdomen revealed liver steatosis, normal spleen with no ascites, and cholelithiasis. Further testing for polycythemia, including JAK2 cascading reflex, BCR-ABL gene mutation, and serum erythropoietin assay, yielded negative results. A peripheral smear of the complete blood picture showed normochromic, normocytic red blood cells. The patient's polycythemia was attributed to possible obstructive sleep apnea with a STOP-BANG score of 4 points, and he was referred to a sleep clinic for further evaluation. However, the patient missed his sleep clinic appointment.

**Figure 1:**
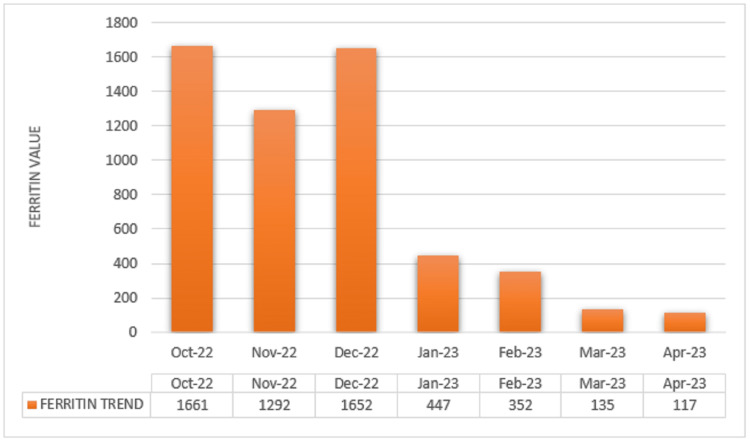
Ferritin level over six months.

**Figure 2:**
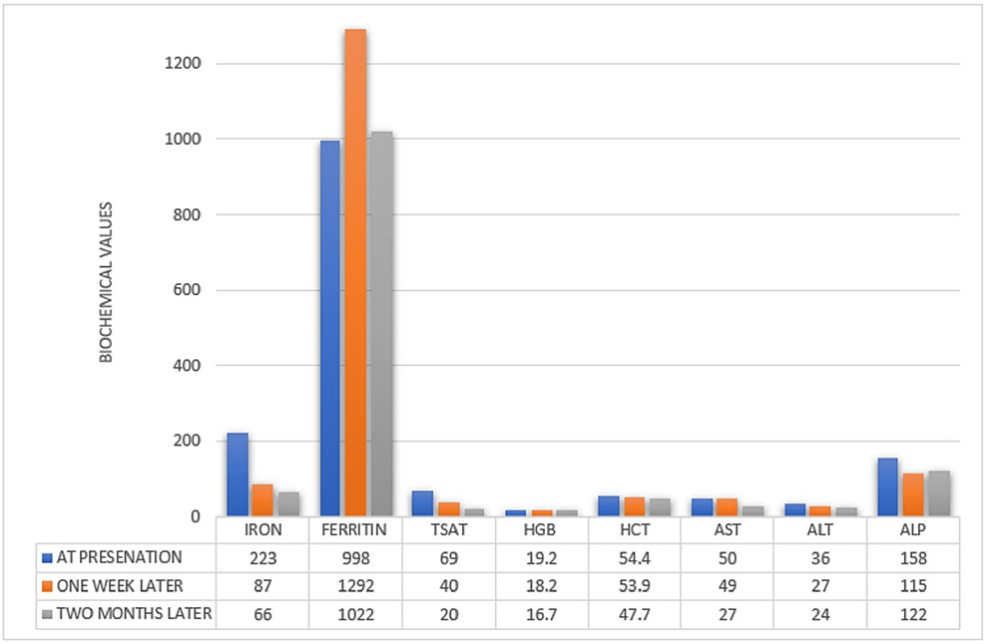
Biochemical trend over two months. AST, aspartate aminotransferase; ALT, alanine aminotransferase; ALP, alkaline phosphatase; Tsat, transferrin saturation; Hgb, hemoglobin; Hct, hematocrit

Elevated ferritin levels along with uncontrolled diabetes mellitus invoked suspicion for hemochromatosis. Genetic testing with polymerase chain reaction (PCR) amplification detected a homozygous mutation for H63D, thereby confirming the diagnosis of HH.Considering the transaminitis, the patient was proposed a liver biopsy to assess potential liver fibrosis. However, approval for charity care is required before proceeding with the biopsy. Hence, a serum liver fibrosis score was obtained, which was negative for fibrosis and inflammation. He was started on therapeutic phlebotomy and encouraged alcohol abstinence. The patient completed eight phlebotomy sessions, resulting in a recent ferritin level of 117 ng/mL, aiming to achieve a target ferritin level of 50 or lower. Figures 1-2 show patients’ responses to treatment.

## Discussion

In primary care, liver enzymes are regularly tested, and elevations are generally found incidentally. Uncontrolled diabetes, transaminitis, and persistently elevated ferritin levels should raise the suspicion of hemochromatosis. HH is an autosomal recessive condition caused by the mutation of the HFE gene on the short arm of chromosome 6, thereby increasing intestinal absorption of iron and iron overload [3]. There are multiple mutations of the HFE gene, but the two most common variants are C282Y and H63D. HH is a common genetic disorder among the Caucasian population, particularly in the Northern European region where the prevalence is 1 per 220 to 250 individuals [1-2]. After years of iron buildup, symptoms usually manifest between 40 and 50 years of age. Males present 10 years earlier than females and have increased ferritin levels. Menstrual blood loss and iron loss during pregnancy have protective effects in females [1].

Pathophysiology

**Figure 3:**
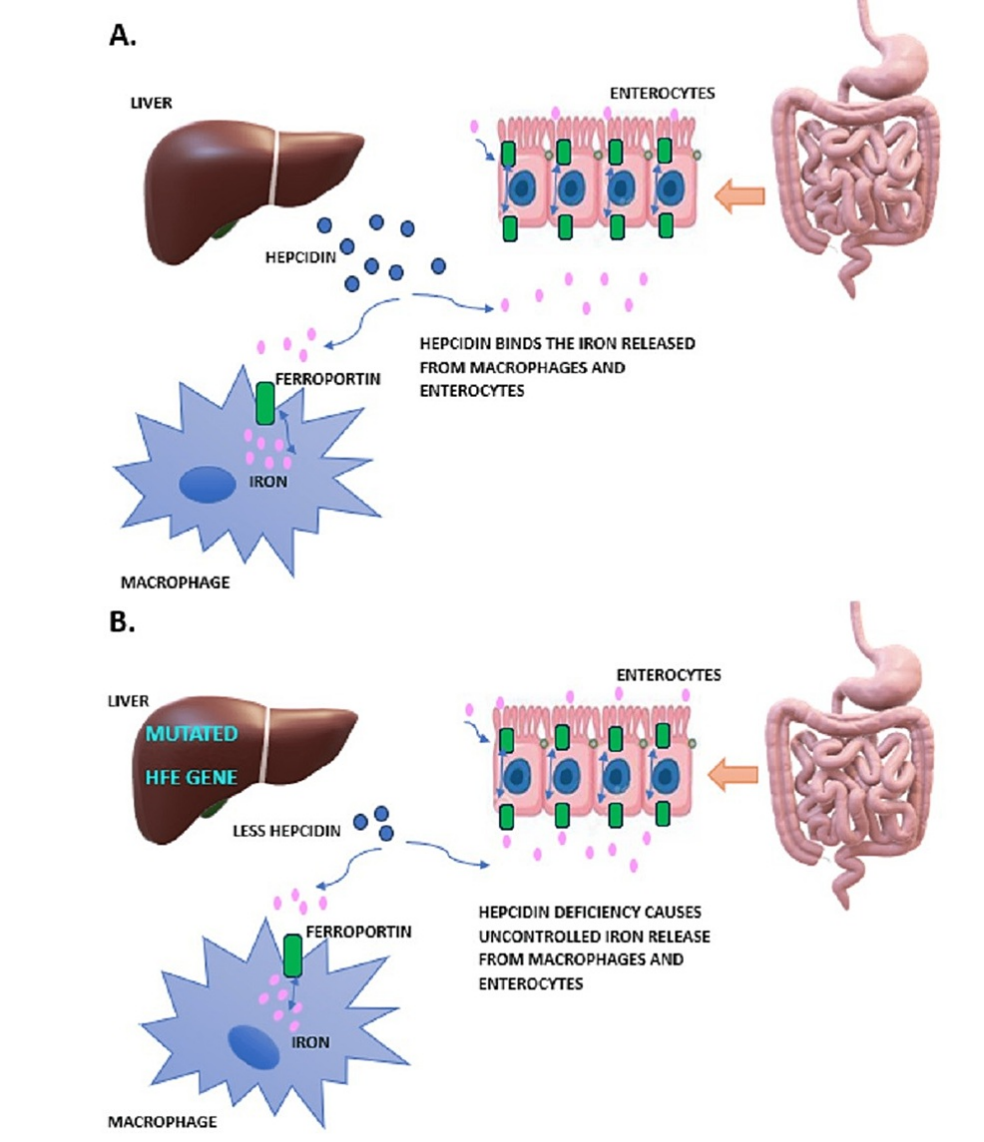
Pathophysiology of hereditary hemochromatosis. Image credit: Venkata Sri Ramani Peesapati. HFE, high iron gene

HH pathology depends on two major proteins, glycoprotein encoded by the HFE gene and transferrin receptor. These two proteins play a major role in inhibiting iron absorption. Iron accumulation occurs through two mechanisms: (1) C282Y hinders the glycoprotein from binding to the transferrin receptor, thereby increasing enteric iron absorption (Figure 3); and (2) decreased generation of hepcidin, which releases iron into the plasma from macrophages and adipocytes (Figure 3). This depletes the intracellular iron stores, further increasing intestinal iron absorption. Non-transferrin-bound plasma iron is absorbed by the liver and causes free radical production, stimulating tissue damage [4,5]. Siderotic necrosis, periportal hepatocellular death due to oxidation of iron-dependent lipids from iron overload, activates macrophages and subsequently causes cirrhosis when the hepatic concentration of iron exceeds 400 mircomol/g [6,7]. This phenomenon also diverts iron deposition into other organ systems such as skin (hyperpigmentation), pancreas (diabetes), heart (cardiomyopathy and arrhythmias), and gonads (hypogonadism).

Clinical features

Common symptoms include weakness, lethargy, arthralgias, and loss of libido. Physical findings of hepatosplenomegaly, symptoms of heart failure, and classical findings of bronze diabetes occur in less than 10% of patients [8]. However, many of the patients are asymptomatic, such as the patient in our case. Routine biochemical analysis reveals elevated ferritin and iron levels prompting further investigation of HH.

Diagnosis

High level of clinical suspicion is required to diagnose HH due to nonspecific signs and symptoms. Serum ferritin levels greater than 200 for women and 300 for men have a sensitivity of 66% and a specificity of 85% [9-11]. In addition, elevated serum ferritin along with increased transferrin saturation levels has a sensitivity of 93% for diagnosing HH [12]. Genetic testing is the mainstay of confirmation for HH; there are combinations of mutations: (1) C282Y homozygote (90% of cases), (2) C282Y/H63D heterozygote, (3) C282Y heterozygote, (4) H63D homozygote (10% of patients, present in our patient), and (5) H63D heterozygote [12]. A hepatic biopsy is used to determine the amount of iron overload and the extent of liver damage. C282Y homozygote genotype in combination with ferritin levels >1,000, elevated AST levels, and hepatomegaly warrants liver biopsy to evaluate for hepatic fibrosis. In contrast, patients with C282Y/H63D heterozygote genotype and ferritin levels less than 500 ng/mL with normal transferrin saturation levels are at relatively low risk for hepatic fibrosis [12].

Treatment

The primary goal of treatment for HH is decreasing preexisting iron deposits through phlebotomy. The amount of blood for extraction is determined using body weight, equating to 7 mL/kg without exceeding a total of 550 mL (equivalent to 250 mg of iron) per session [13]. Ferritin and transferrin levels are measured for depletion confirmation. Generally, ferritin levels of more than 50 mg/mL and normal transferrin saturation levels are affirming, and four to eight maintenance phlebotomy sessions are required to maintain these levels [14-16]. Iron accumulation varies among patients; thus, the frequency of phlebotomy sessions is tailored to each individual's needs. It is advisable to conduct follow-up assessments every two to three months for the purpose of reevaluating ferritin levels [2].

## Conclusions

Abnormal liver function serology can be incidental in primary care settings and needs a thorough serological evaluation to rule out viral or metabolic etiology. Iron studies, as a part of metabolic workup, are performed to evaluate for hemochromatosis. HFE gene testing can establish the diagnosis of hemochromatosis, while liver biopsy holds prognostic significance. Since liver cirrhosis determines prognosis and survival in patients with HH, early diagnosis and treatment with phlebotomy are critical to mitigate the risk of cardiohepatic dysfunction and to extend the overall lifespan. 
